# Supplementary material for: An Assay System to Evaluate Riboflavin/UV-A Corneal Phototherapy Efficacy in a Porcine Corneal Organ Culture Model
Source: Animals (Basel). 2020 Apr 23;10(4):730. doi: 10.3390/ani10040730 (PMC7652214; doi:10.3390/ani10040730)
Supplement: Supplementary file 1 [file animals-10-00730-s001.zip › Statistical Analysis - supplementary materials-revised version.doc]

Appendix - *Statistical modelling and data analytics*

In order to formalize the comparison among the three populations under investigation, i.e., healthy, injured, and treated, we assume an ANOVA-type multi-way experimental data representation model. More formally and without loss of generality, let us assume that the brightness Y, our damage-related histological measure, measured on the *i*-th slide (our experimental unit), from the *l*-th subject belonging to the *j*-th population, at the *s*-th ROI (region of interest—each of the 24 combinations of the 4 depth layers and the 6 radial frames), can be modelled as:

Y*_ilsj_* = µ + τ*_j_* + β*_s_* + (τβ)*_js_* + ε*_ilsj_*, (1)

where ε*_ilsj_* are i.i.d. possibly non-Gaussian error terms with null mean and scale coefficients *σ_j_*^2^=*σ*^2^(τ*_j_*) and unknown distribution *P****_ε_***; µ is a population-invariant constant; coefficients τ*_j_* represent the main population effects; β*_s_* is the ROI effect; (τβ)*_js_* is the interaction between population and ROI effects; and *σ*^2^(τ*_j_*) are population-varying scale coefficients which may depend, through monotonic functions, on main treatment effects τ*_j_*. Basically, the proposed data representation model is generally a less-demanding nonparametric model where specific location and scale effects are both allowed across populations.

Since the study’s main goal is to compare the corneal populations, we actually inferred the main population coefficients τ*_j_*. In order to retain a flexible and reliable inferential analysis, we applied a nonparametric testing approach; thus, we proposed a suitable extension to Equation (1) of the nonparametric combination NPC testing method (Bonnini et al., 2014).

For this goal, let us formalize the comparison between the *j*-th and the *h*-th populations separately for the location and scatter parameters via Roy’s Union-Intersection testing approach (Pesarin and Salmaso, 2010), stating the null and alternative hypotheses as follows:

$\left\{ \begin{matrix} H_{0\left( jh \right)}:\bigcap_{s}Y_{sj}{}_{=}^{loc}{Y_{sh}}\equiv\bigcap_{s}\left[ \tau_{sj}=\tau_{sh} \right] \\ \begin{matrix} \\ H_{1\left( jh \right)}:\bigcup_{s}\left[ \left( Y_{sj}{}_{<}^{loc}{Y_{sh}} \right)\bigcup\left( Y_{sj}{}_{>}^{loc}{Y_{sh}} \right) \right] \end{matrix} \\ \equiv\bigcup_{s}\left[ \left( \tau_{sj}<\tau_{sh} \right)\bigcup\left( \tau_{sj}>\tau_{sh} \right) \right] \end{matrix} \right.$ $\left\{ \begin{matrix} H_{0\left( jh \right)}:\bigcap_{s}Y_{sj}{}_{=}^{sca}{\tilde{Y}_{sh}}\equiv\bigcap_{s}\left[ \sigma_{sj}^{2}=\sigma_{sh}^{2} \right] \\ \begin{matrix} \\ H_{1\left( jh \right)}:\bigcup_{s}\left[ \left( Y_{sj}{}_{<}^{sca}{Y_{sh}} \right)\bigcup\left( Y_{sj}{}_{>}^{sca}{Y_{sh}} \right) \right] \end{matrix} \\ \equiv\bigcup_{s}\left[ \left( \sigma_{sj}^{2}<\sigma_{sh}^{2} \right)\bigcup\left( \sigma_{sj}^{2}>\sigma_{sh}^{2} \right) \right] \end{matrix} \right.$ (2)

where τ*_sj_* =τ*_j_* + (τβ)*_js_* *s* = 1,…,24, is the reference index for each region of interest.

It is worth noting that hypotheses of Equation (2) refer to a multistrata version of the so-called generalized Beherens–Fisher problem (Yanagihara and Yuan, 2005), which is a difficult testing problem to handle within traditional parametric methods (Pesarin and Salmaso, 2010). Under the null hypothesis, those observed data are approximately exchangeable random components that can be permuted between groups in order to derive, separately for the location and scatter problems, two combined overall ROI directional *p*-values.

As univariate location and scatter test statistics we respectively used the differences of sample means and squared deviations together with, as a combining function to derive the overall *p*-values across all ROIs, Fisher’s combining function. For a more in-depth understanding of the testing procedure outlined here, we refer readers to Corain and Salmaso (2015).

By exploiting the combined across-ROI one-sided alternatives in Equation (2), we may derive two location and scatter rankings using the ranking methodology proposed by Arboretti et al. (2014). In fact, by suitable combining information from directional *p*-values, the underlying latent ordering among τ*_j_* and *σ_j_*^2^ parameters can be properly estimated. In a nutshell, the rationale behind the ranking within a multivariate setting is the following: if not all $H_{0(jh)}$ in Equation (2) are true, there must exist an ordering [1],[2],…,[*C*] among τ*_j_* and *σ_j_*^2^ such that:

τ_[1]_≤τ_[2]_≤…≤τ_[_*_C_*_]_ and *σ*^2^_[1]_≤*σ*^2^_[2]_≤…≤*σ*^2^_[_*_C_*_]_

For more details on the ranking methodology we refer the reader to Arboretti et al. (2014), and Corain et al. (2016).
