# Supplementary figures and images for: An Assay System to Evaluate Riboflavin/UV-A Corneal Phototherapy Efficacy in a Porcine Corneal Organ Culture Model
Source: Animals (Basel). 2020 Apr 23;10(4):730. doi: 10.3390/ani10040730 (PMC7652214; doi:10.3390/ani10040730)

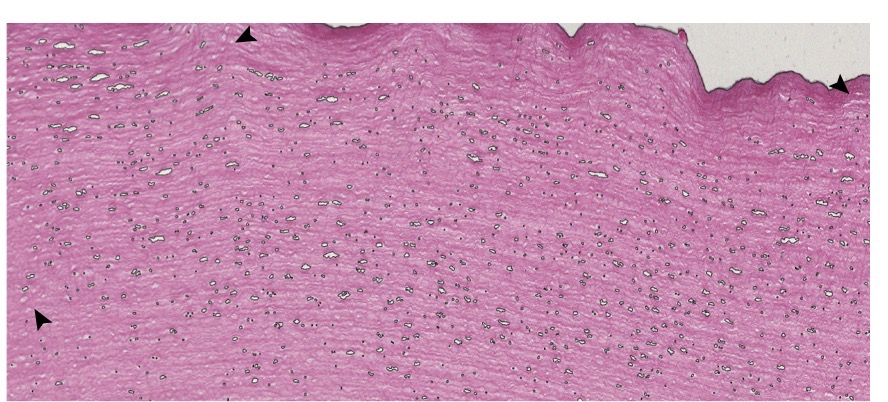

Supplement: Supplementary file 1 [file animals-10-00730-s001.zip › Supplementary Figure 2.tiff]

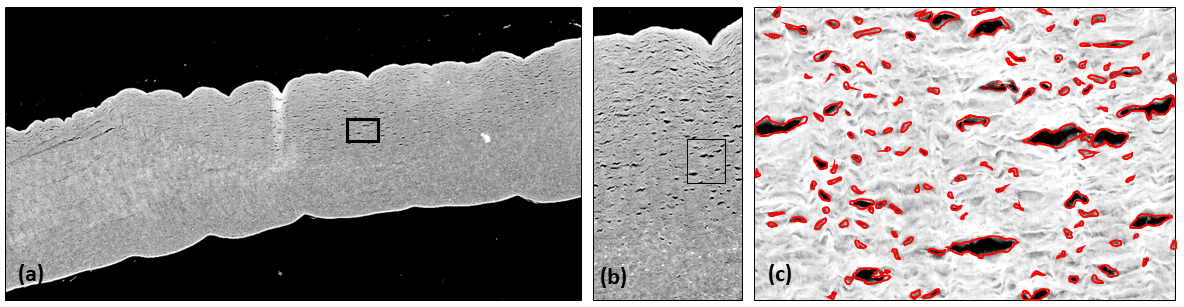

Supplement: Supplementary file 1 [file animals-10-00730-s001.zip › Supplementary Figure 1.tif]
